# Efficient artificial intelligence-based assessment of the gastroesophageal valve with Hill classification through active learning

**DOI:** 10.1038/s41598-024-68866-x

**Published:** 2024-08-13

**Authors:** Ioannis Kafetzis, Karl-Hermann Fuchs, Philipp Sodmann, Joel Troya, Wolfram Zoller, Alexander Meining, Alexander Hann

**Affiliations:** 1 Interventional and Experimental Endoscopy (InExEn), Department of Internal Medicine 2, University Hospital Würzburg, Oberdürrbacherstr. 6, 97080 Würzburg, Germany; 2 Clinic for General Internal Medicine, Gastroenterology, Hepatology and Infectiology, Pneumology, Klinikum Stuttgart–Katharinenhospital, Kriegsbergstr. 60, 70174 Stuttgart, Germany

**Keywords:** Active learning, Artificial intelligence, Gastroscopy, Hill classification, Hiatal hernia, Gastroenterology, Gastrointestinal diseases

## Abstract

Standardized assessment of the gastroesophageal valve during endoscopy, attainable via the Hill classification, is important for clinical assessment and therapeutic decision making. The Hill classification is associated with the presence of hiatal hernia (HH), a common endoscopic finding connected to gastro-esophageal reflux disease. A novel efficient medical artificial intelligence (AI) training pipeline using active learning (AL) is designed. We identified 21,970 gastroscopic images as training data and used our AL to train a model for predicting the Hill classification and detecting HH. Performance of the AL and traditionally trained models were evaluated on an external expert-annotated image collection. The AL model achieved accuracy of 76%. A traditionally trained model with 125% more training data achieved 77% accuracy. Furthermore, the AL model achieved higher precision than the traditional one for rare classes, with 0.54 versus 0.39 (*p* < 0.05) for grade 3 and 0.72 versus 0.61 (*p* < 0.05) for grade 4. In detecting HH, the AL model achieved 94% accuracy, 0.72 precision and 0.74 recall. Our AL pipeline is more efficient than traditional methods in training AI for endoscopy.

## Introduction

Hiatal hernia (HH) is a common finding in upper gastrointestinal endoscopy in patients suffering from foregut symptoms^1,2^. Controversy exists regarding its endoscopic assessment, classifications, and relation to gastroesophageal reflux disease and chronic anemia^3^. In general, HH can be defined as migration of the stomach, esophago-gastric-junction, and rarely other visceral organs, into the mediastinum, in the setting of deterioration of the phrenoesophageal ligament and widening of the hiatus.

Several classifications attempting to standardize the assessment HH based on various anatomical alterations have been introduced^4,5^ and thoroughly reviewed^6^. The only endoscopy-based classification is the Hill classification, describing the status of the flap valve at the cardia and hiatus^7^. This grading is endoscopically determined via visual inspection of the flap valve during retroflexion in gastroscopy. It has been shown to have significant clinical relevance, as it is connected to esophagitis^8^, development of postoperative GERD and erosive esophagitis after laparoscopic sleeve gastrectomy^9^, and it is considered superior to axial length for assessing the antireflux barrier^10^. Furthermore, image documentation of the valve inspection is included in multiple guides as part of the standard gastroscopy reporting procedure in multiple guidelines^11–13^.

The visual nature of the Hill classification makes utilizing artificial intelligence (AI) methods for its prediction feasible. This is further motivated by the increasing introduction of AI in the medical field^14,15^ and especially endoscopy^16–18^ with applications including polyp detection^19–21^, polyp size estimation^22^, and examination report generation^23^. Focusing on gastroscopy, AI has found applications in several problems, such as gastric polyp detection^24^, diagnosis of atrophic gastritis^25^, real-time image reporting^26^, and Barretts’ neoplasia detection and delineation^27,28^. AI has been utilized to diagnose HH, based on color space transformations and histogram normalization of endoscopic images^29^ and decision tree models from bariatric data^30^. Another approach utilized AI methods to diagnose HH through chest radiographs^31,32^. To our knowledge, no AI-based method for predicting the Hill classification exists.

As AI is already implemented and used as a valorous tool in clinical practice^16^, improving efficiency of AI training is essential, as quality labeled data can only be obtained through expert annotations. However, medical experts usually have limited time and are often expected to annotate copious amounts of data at once. This approach can lead to tiredness, lack of motivation, and stress, all of which are factors that diminish the quality of annotations^33^. To improve AI-training efficiency, we implemented a novel active learning (AL)^34^ pipeline, which works multiple steps that can be undertaken at the expert's own pace. Using AL, we trained an AI for predicting the Hill classification and through its predictions infer the presence of HH. The proposed AL-pipeline is shown to result in higher performing models, compared to traditional AI training.

## Methods

### The Hill classification

**Figure 1:**
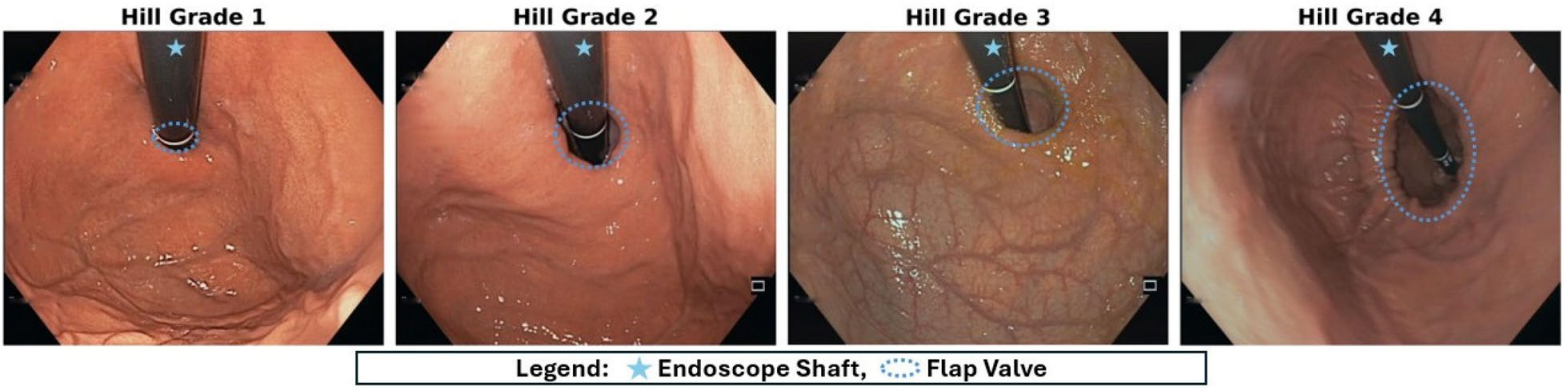
Examples of endoscopic images of flap valve inspection captured during retroflexion in the stomach, depicting the four different Hill grades.

The Hill grade is a classification of the antireflux barrier that focuses on the valve. Hill differentiated four different grades, described next. A grade 1 flap valve has the ridge of the tissue at the cardia preserved and closely approximated to the shaft of the retroflexed scope, extending 3–4 cm along the lesser curvature, which is considered a normal physiologic situation. A grade 2 flap valve has a less pronounced ridge at the cardia, which may open with respiration. A grade 3 flap valve describes a diminished ridge of the cardia along with failure to close around the endoscope. A grade 4 flap valve: the muscular ridge at the cardia is absent; the esophago–gastric junction stays open and the endoscopist may easily view the esophageal lumen in retroflexion. Also, Hill classification has been shown to be connected to HH, with Hill grades 3 and 4 associated with its presence and grades 1 and 2 with its absence. Examples of the different Hill classes are depicted in Fig. 1.

### Train and test datasets

Data used for AI training consisted of retroflexion images captured during routine gastroscopy. 71,877 examinations, performed in two hospitals from 2015 to 2021, were screened. Out of these, 46,068 were excluded due to incomplete documentation, where no image data associated with the examination existed. A further 10,926 examinations were excluded because they lacked images in retroflexion. The final training data consisted of 21,970 images from 14,883 gastroscopies.

**Figure 2:**
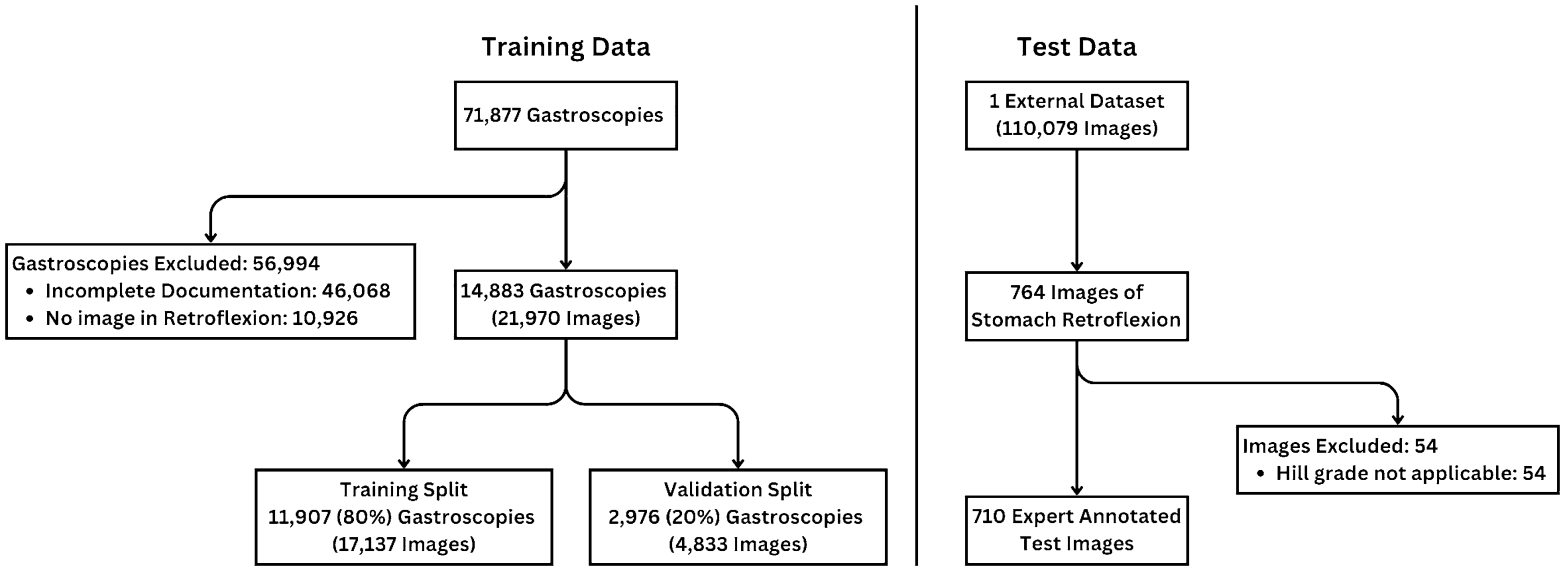
Visualization of training (left) and testing (right) data. The training data was obtained from a collection of examination image reports. Test data consisted of images from an external dataset of endoscopic images.

To prevent data leakage, the images were split into training and validation on examination level, such that if multiple images from the same examination were selected, they were all used exclusively for training or validation. Following an 80–20% train-validation split resulted in 11,907 examinations with 17,137 images for training and 2976 examinations, with 4833 images, for validation. The trained AI models were evaluated on images from an external dataset of endoscopic images, HyperKvasir^40^. This dataset contains multiple images of anatomical landmarks from the upper and lower gastroesophageal tract. Out of these, the 764 images from the “retroflex-stomach” category were presented to the expert endoscopist, with over 30 years of experience in endoscopy, and specialized on the diagnosis and different treatment modalities of gastroesophageal reflux disease, for annotation. The expert evaluated if the Hill classification was applicable for each image, and assessed the Hill grade when it was. The final test dataset contained 710 images with their corresponding Hill grade. The distribution of Hill labels in the test data was 368 (51.8%) grade 1, 257 (36.2%) grade 2, 67 (9.4%) grade 3, and 18 (2.5%) grade 4. Generation of the training, validation and test datasets is presented in Fig. 2.

### Proposed active learning pipeline

AL has found successful applications in the medical field^41–44^ for many medical computer vision tasks such as image classification^45,46^ and segmentation^47,48^. It proves beneficial under class imbalance, a common issue in medical data, where examples of one or more classes are sparse in the dataset, making them difficult to find using random selection.

Active learning uses a large set of unlabeled data, usually called a pool, and an AI model for the task at hand. In traditional AI training, unlabeled data are annotated in random order. The goal of active learning is to optimize the selection process for annotation data, by utilizing AI predictions. Through them, unlabeled data that can improve model training the most are selected for annotation. After annotating the selected data, a new, improved model is trained, and its predictions are then used for selecting further data points for annotation. In more detail, model predictions are initially obtained for all unlabeled data. These predictions are used with a selection method, called acquisition function, which selects the most information rich subset of the unlabeled data to be annotated^42^. The expert annotates the selected data, and a new model is trained using all annotated data. Active learning is the repetition of these steps, where the AI trained at each step is used to predict on the remaining unlabeled data. The acquisition function is the heart of AL pipeline^49^, as it is responsible for selecting data points to be included in the training of subsequent models. In traditional AI training data points are selected at random and therefore random selection serves as a baseline for evaluating AL.

This work proposes an AL pipeline based on a novel acquisition function that results in a diverse and representative set of annotated data. Data are selected for each label sequentially. The predictions for each label were split into 10 bins of equal length. Initially, a non-empty bin was randomly uniformly selected. Subsequently, an image from the selected bin was randomly selected for annotation. The first part of the selection enabled inclusion of images for annotation that varied from easy to hard examples, thus resulting in a representative dataset.

In this work, the AL iterations occurred until 10% (2400 images) of the total unlabeled data were annotated. At each AL iteration, 300 images, 75 per label, were selected for annotation, for 8 iterations. To enable comparison with traditional training, the same process but with 300 randomly selected data each iteration was used as a baseline.

The pipeline and the trained model are made publicly available, empowering physicians to train their own AI models without requiring programming skills https://www.ukw.de/en/inexen/code-data-and-3d-models/﻿.

### Model architecture and training

All AI models trained followed the same architecture, using a ConvNext^50^, pre-trained on ImageNet^51^, as backbone, followed by a fully connected layer with 4 output neurons one for each Hill grade. Predictions were obtained by applying the SoftMax activation to the model output and selecting the label with the highest value as the predicted one. At each active learning step, a new pre-trained model was initialized and fine-tuned from scratch. The optimizer used in model AdamW, with the loss function was the standard cross entropy. The model saved at each AL step was the one achieving the lowest loss on the validation set over 50 epochs.

To improve interpretability of model predictions, the Grad-CAM (Gradient-weighted Class Activation Mapping) algorithm was used^52,53^. The Grad-CAM algorithm generates a heatmap that highlights the image regions contributing the most to the model’s classification decision.

### Evaluation

The annotation protocol used for evaluating the proposed AL pipeline compared to traditional AI training was as follows. Initially, the same 21,970 unlabeled images, identified as potential training and validation data. For traditional training, images were sequentially sampled from the available data and presented to the expert for annotation, as is standard in AI training. Models were trained with predetermined amounts of sequentially selected, annotated images, namely 2400 and 5400 images, to enable comparison with the AL model. For the AL, starting from the same original data pool of 21,970 unlabeled images, 300 images were identified using model predictions at each step and presented to the expert for annotation. These images were used to train a new AI, the predictions of which were used in the next unlabeled image selection round. This process continued until a predefined limit of 2400 images, about 10% of the unlabeled dataset, was annotated. In all cases, annotations were provided by an expert endoscopist with over 30 years of experience. The expert was presented with each image and was asked to provide one classification label for each image, indicating the Hill grade, or if the image is not sufficient for assessing the Hill grade, thus excluding it from training.

Model performance was evaluated in terms of assessing the Hill classification. Each image of the test dataset was presented to the expert endoscopist for annotation, who again provided one label for each image, indicating either the Hill grade for the image or that the Hill classification is not applicable with the current image. The label provided by the expert endoscopist was the gold standard used to assess model performance. Images where the expert deemed insufficient to assess with the Hill grade were excluded from model evaluation. Special attention was given to accuracy, precision, recall (sensitivity), and specificity for each grade individually. The AL pipeline was assessed comparing the mean per-class accuracy, precision, recall (sensitivity), and specificity of models trained using the same number of images, selected either with our AL pipeline or with the traditional method. Furthermore, the distribution of the different grades in images with the two methods was compared. Furthermore, the last AL model was compared with a model trained with the traditional method, using 225% the training data, 2400 for AL versus 5400 for traditional training. The ability to infer the presence of HH (Hill grades 1–2 vs. grades 3–4) was assessed with accuracy, precision, recall (sensitivity), and specificity. The 95% confidence intervals were obtained via bootstrapping and statistical differences were investigated with the *t* test. To enable comparison with existing approach in diagnosing HH, the values for specificity and sensitivity were also calculated.

## Results

The model trained with the AL pipeline, after the pre-defined termination condition of 2400 images was met, achieved an accuracy of 76% in assessing the Hill classification, compared to the 73% achieved by the traditionally trained model with the same number of images. In the per-class analysis, the AL model demonstrated greater or equal accuracy with its traditional counterpart for all classes. For the rare classes, the AL model demonstrated higher precision, with 0.54 (95% CI 0.42–0.66) versus 0.34 (95% CI 0.23–0.46) for grade 3 and 0.72 (95% CI 0.50–0.92) versus 0.56 (95% CI 0.31–0.80) for grade 4, while maintaining high performance in terms of recall (sensitivity), with 0.58 (95% CI 0.46–0.71) versus 0.56 (95% CI 0.40–0.71) for grade 3 and 0.52 (95% CI 0.32–0.72) vs 0.62 (95% CI 0.38–0.87) for grade 4. The AL model was more specific for the common grade 1 with 0,88 (95% CI 0.84–0.91) versus the 0.82 (95% CI 0.77–0.86) of the traditional model for the same grade.

**Figure 3:**
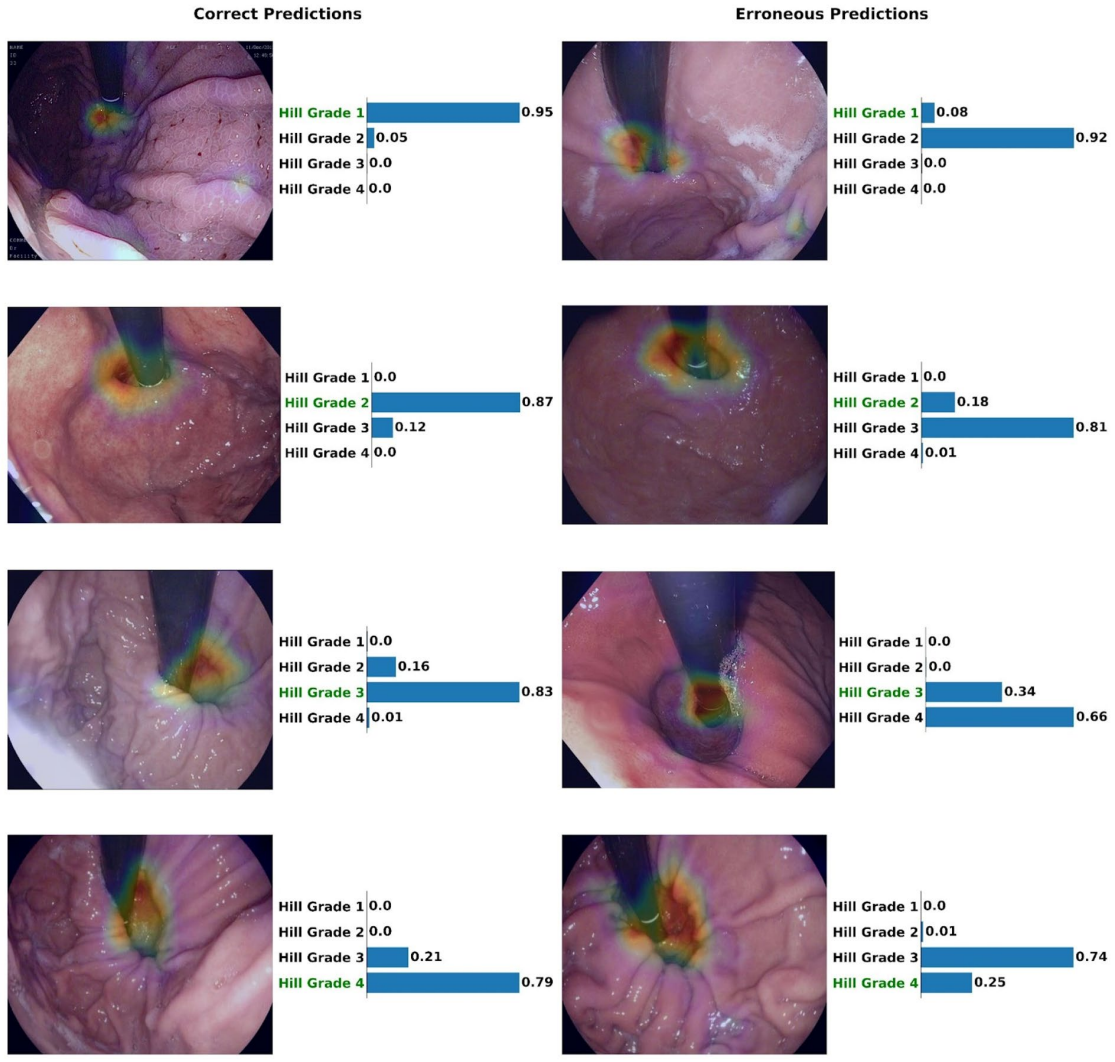
Visualization of Grad-CAM results and prediction probabilities for correctly and erroneously classified images for the four different Hill grades. The results correspond to images from the distinct test dataset^40^. Hill grade with green colored letters indicates the gold standard label, and bars indicate model predictions.

A traditionally trained model for the same task, with 5,400 images, that is, with 125% more training data used for the AL model achieved accuracy of 77%. Even with this amount of data, the AL model was more precise, with 0.54 (95% CI 0.42–0.66) versus 0.39 (95% CI 0.27–0.51) for grade 3 and 0.72 (95% CI 0.50–0.92) versus 0.61 (95% CI 0.36–0.84) for grade 4. The extended amount of data allowed the traditional model to demonstrate a higher specificity of 0.90 (95% CI 0.86–0.93) for grade 1, which is a mere improvement of 0.02 from the 0.88 (95% CI 0.84–0.91) that the AL model achieved for the same grade. The complete per-class analysis is reported in Table 1. Furthermore, the GradCAM method results were collected for correct and erroneous assessments of the Hill grade. Examples of such assessments on images from the external test dataset, including the explainability heatmap and prediction confidences for each class are depicted in Fig. 3.

**Table 1:** Per Hill grade evaluation of models on the external test data.

	2400 images		5400 images
	Active learning	Traditional training	Traditional training
Grade 1			
Accuracy (95% CI)	83% (80–86)	81% (78–84)	85% (82–87)
Precision (95% CI)	0.90 (0.87–0.93)	0.83 (0.80–0.87)	0.92 (0.89–0.95)
Recall (Sensitivity) (95% CI)	0.79 (0.75–0.83)	0.81 (0.77–0.85)	0.81 (0.77–0.84)
Specificity (95% CI)	0.88 (0.84–0.91)	0.82 (0.77–0.86)	0.90 (0.86–0.93)
Grade 2			
Accuracy (95% CI)	79% (76–82)	75% (72–78)	79% (76–82)
Precision (95% CI)	0.61 (0.55–0.67)	0.69 (0.63–0.75)	0.66 (0.60–0.72)
Recall (Sensitivity) (95% CI)	0.76 (0.70–0.82)	0.65 (0.59–0.71)	0.74 (0.68–0.79)
Specificity (95% CI)	0.80 (0.76–0.84)	0.82 (0.78–0.85)	0.81 (0.78–0.85)
Grade 3			
Accuracy (95% CI)	92% (90–94)	91% (89–93)	92% (90%–94%)
Precision (95% CI)	0.54 (0.42–0.66)	0.34 (0.23–0.46)	0.39 (0.27–0.51)
Recall (Sensitivity) (95% CI)	0.58 (0.46–0.71)	0.56 (0.40–0.71)	0.62 (0.47–0.77)
Specificity (95% CI)	0.95 (0.94–0.97)	0.93 (0.91–0.95)	0.94 (0.92–0.96)
Grade 4			
Accuracy (95% CI)	98% (96–99)	98% (97–99)	98% (97–99)
Precision (95% CI)	0.72 (0.50–0.92)	0.56 (0.31–0.80)	0.61 (0.36–0.84)
Recall (Sensitivity) (95% CI)	0.52 (0.32–0.72)	0.62 (0.38–0.87)	0.65 (0.40–0.88)
Specificity (95% CI)	0.99 (0.99–1.00)	0.99 (0.98–1.00)	0.99 (0.98–1.00)

The active learning model (first column) trained with 2400 images is compared to traditionally trained model with the same (column 2) and 225% training data (column 3). CI: Confidence interval.

**Figure 4:**
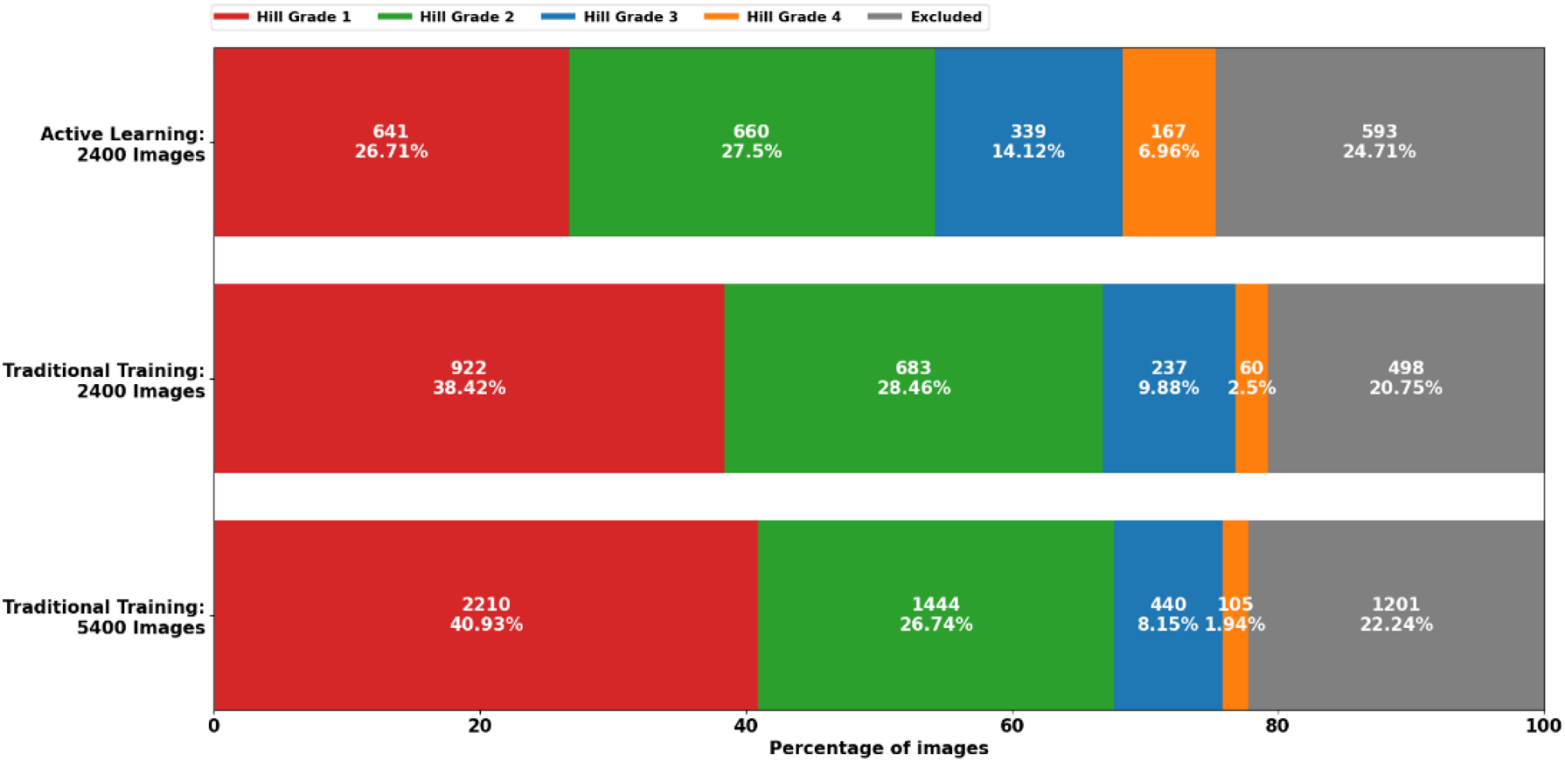
Distribution of the different labels in the annotated data based on our active learning with 2400 images (first row) and traditional training with 2400 (second row) and 5400 images (third row). Each label is represented with a different color. Images excluded from training (assessed as irrelevant or low quality from the expert) are presented in gray.

This performance difference can be attributed to the ability of the AL pipeline to select data from under-represented classes for annotation. Out of the 2400 selected images from the AL pipeline 339 (14.1%) were grade 3 and 167 (7.0%) were grade 4. In traditional training, for the same number of total images 237 (9.9%) were grade 3 and 60 (2.5%) were grade 4. Even after 5,400, traditional training collected 440 (8.1%) images with grade 3 and 105 (1.9%) images of grade 4. The distribution of all labels in the training dataset is depicted in Fig. 4. Model predictions together with the Grad-CAM heatmaps on sequential video frames of flap-valve inspection during gastroscopy for the different Hill grades are depicted in Supplementary Video 1.

In diagnosing HH (Hill grades 1–2 vs. 3–4) the AL model achieved 94% (95% CI 92–95) accuracy, 0.72 (95% CI 0.64–0.80) precision, 0.74 (95% CI 0.66–0.82) recall (sensitivity), and specificity of 0.96 (95% CI 0.95–0.98). The traditional trained model, trained with the same number of images achieved 93% (95% CI 91–95) accuracy, 0.81 (95% CI 0.72–0.89) precision but 0.54 (95% CI 0.47–0.63) recall (sensitivity), and 0.98 (95% CI 0.97–0.99) specificity. Similarly, the traditionally trained model trained with 125% more selected images compared to of the AL achieved accuracy of 94% (95% CI 92–95) percent, 0.85 (95% CI 0.77–0.92) precision but 0.59 (95% CI 0.50–0.68) recall (sensitivity) and 0.99 (95% CI 0.98–0.99) specificity.

## Discussion

The potential benefits of AI in medicine are being thoroughly investigated. In endoscopy, multiple commercially available AI solutions exist and are implemented in clinical routine to support physicians during the examination. Developing effective medical AI required, in most cases, expert annotated data, which is a limiting factor as experts usually have limited time. Furthermore, a common pitfall for medical data is class imbalance, which describes the existence of data classes that are significantly less represented. Traditional AI training selects data for annotation randomly, thus resulting in significantly lower chance for data from rare classes to be selected^35^. Therefore, the need for generation of efficient AI training methods, that account for class imbalance and can be undertaken at the expert's pace is imminent.

Several works have attempted to solve the above problems using active learning, where the idea is to stratify how images are selected for annotation, using the predictions of an existing, weak AI model. Most existing AL pipelines select data for which the model is most “uncertain”, as these are more likely to result in erroneous predictions. Examples of selection methods include choosing the least confidence predictions^36^, highest Shannon entropy^37^ and marginal sampling^38^. These methods tend to select “hard” examples, which may not be representative of the original data.

This work proposes a novel AL pipeline that accounts for rare classes, resulting in a diverse and representative collection of annotated data. The novelty of the proposed AL method lies in how images are selected for annotation. Instead of selecting randomly, or focusing on hard examples, the method uniformly selects data from the entire range of model predictions, enabling selection of easy to hard positive and negative examples. The above selection generates a diverse set of annotated examples. Furthermore, uniformity in the selection enables more images from under-represented classes to be included in the training dataset for the model.

We applied the proposed pipeline for training an AI model that predicts the Hill classification in gastroscopy. The Hill classification assesses the status of the gastroesophageal valve by assigning a grade, from 1 to 4. Grades 3 and 4 are rare in the screening population from which the training data come from.

21,970 unlabeled images captured during gastroscopy were identified as training data. The stopping criterion for the AL was set to 2400 when 10% of the training data were annotated. Additionally, we used traditional AI training as the baseline, and compared the AL trained model with traditionally trained model with 2400 and 5400 images. Out of 2400 images, using the AL pipeline resulted in 339 (14.1%) grade 3 and 167 (7.0%) grade 4 data points. In a total of 2400 images, 237 (9.9%) represented grade 3 and merely 60 (2.5%) grade 4. Traditional training with 225% data, that is 5400 images, found 440 (8.1%) grade 3 images and 105 (1.9%) images of grade 4, which are still less of what AL managed to obtain. This demonstrates that the AL method generates a diverse dataset including more examples of rare classes, allowing training of more effective models.

The data selection method's efficiency is shown in the performance of the trained models, which were evaluated on expert-annotated images from a distinct set of images from an external dataset of endoscopic images. The AL trained model achieved a 76% accuracy in assessing the Hill grade compared to the 71% of its traditional trained counterpart. Traditional training, with 225% of the data, managed to improve accuracy to 77%. This demonstrates that traditional training requires a larger volume of data to achieve similar performance. This becomes more evident when considering the per-class analysis, specifically for rare classes. The AL and traditional model achieve comparable, high performance in terms of accuracy and specificity for grades 3 and 4. The major difference comes in precision, where for grades 3 and 4 the AL model achieves 0.54 and 0.72, whereas the traditional model achieves a mere 0.34 and 0.56. This vast difference can be attributed to the selection method, as the rare classes are better represented and a greater percentage of the training data for the AL model. The increased number of examples renders the AL model more effective in identifying these classes. When the Hill classification is used to infer the presence of HH, grades 3 and 4 are associated with HH whereas grades 1 and 2 are associated with its absence. The accuracy, precision, recall (sensitivity), and specificity were 94%, 0.72, 0.74, and 0.96 for the AL model and 93%, 0.81, 0.54, and 0.98 for the traditional model. For HH, the AL model presents a much higher recall, which emphasizes its ability to detect cases of HH that would be missed from the traditionally trained model. Overall, AL developed demonstrated powerful performance which is improved compared to its traditional counterpart. Explainability of the AL model for correct and erroneous examples was investigated using the GradCAM method, which demonstrated that the model focuses on the correct parts of the image when predicting. Erroneous predictions were attributed to erroneous assessment of the size of the flap valve from the model.

The AI models trained in this work achieved a mean accuracy of 76% for the different Hill grades, which can be less than that of expert physicians, yet model performance can improve by continuing model training. Furthermore, we believe that the model can support the decision process for younger physicians with limited experience and in the automation of the examination reporting process.

Despite the significance of the Hill classification, to our knowledge, the problem of automating its determination using AI has not been addressed so far. Regarding the detection of HH, comparison of our results with previous works involving gastroscopic images^29^ or patient data from bariatric interventions^30^ presents that our model achieves similar outcomes and a smaller gap between specificity and sensitivity (Table 2). Thus, the obtained model was able to infer the presence HH robustly. Similarly, Santeramo et al.^32^ utilized deep learning methods for chest radiographs to predict the existence of several abnormalities, including HH.

**Table 2:** Overview of performance measures for detection of hiatal hernia by different models compared to our work.

Model	Sensitivity	Specificity	Accuracy
Serpa-Andrade et al.^29^			
CIELab + KNN	0.81	0.60	0.77
CIELab + RF	0.87	0.64	0.83
LBP-FD + KNN	0.94	0.40	0.83
LBP-FD + RF	0.83	0.60	0.81
Assaf^30^			
Decision tree	0.40	0.88	0.85
Decision tree + BS	0.47	0.92	0.88
Our AL model	0.74	0.96	0.94

Our work presents different limitations. One could also argue that annotating in batches is not convenient for the expert, as the annotation process is interrupted by model training. Yet, this fact proves beneficial when attempting to integrate annotation processes in the medical routine, for example between examinations^39^. Furthermore, a limitation is that the model was trained and tested on static images, instead of video frames which could result in the model not performing the same during an examination Yet, frame-by-frame application of the model in examination videos demonstrated robust performance, which makes us confident in the ability of the model to be compatible with real-time application during clinical routine. One limitation of this study is that the test dataset contained fewer images for Hill grades 3 and 4 compared to grades 1 and 2. This imbalance may affect the accuracy of model evaluation, particularly in larger datasets. However, it is noteworthy that an analogous distribution of Hill grades is expected in the general population undergoing screening gastroscopies. Therefore, the analysis of model performance remains relevant and fitting for this clinical scenario. While the model obtained demonstrates promising results overall, there are areas for improvement, particularly in outcomes for Hill grades 3 and 4. Model performance in these critical scenarios can be enhanced by increasing the number of images used in model training. This, as was shown in this work, can be efficiently done by continuing the AL based training. This work introduces a novel AL pipeline that enables efficient training of AI models, especially under class imbalance. The training process is iterative, and the annotation process can fit the time availability of experts providing labels. Using the proposed pipeline, we trained AI for predicting the Hill classification from gastroscopic images and infer the presence of hiatal hernia. The model was evaluated on an external set of expert-annotated images and its performance was compared to that of traditionally trained AIs. Both the AL pipeline and trained model checkpoints are made publicly available and can be downloaded from https://www.ukw.de/en/inexen/code-data-and-3d-models/.

### Supplementary Information

Supplementary Video 1.

## Data Availability

Training data is not made publicly available as it contains sensitive patient information. Test image data are available from the HyperKvasir dataset (10.6084/m9.figshare.12759833). Expert annotations for the test data can be provided by reasonable request to the corresponding author.
